# Changes in Dendritic Architecture: Not Your “Usual Suspect” in Control of the Onset of Puberty in Male Rats

**DOI:** 10.3389/fendo.2013.00078

**Published:** 2013-06-28

**Authors:** Peter J. Hemond, Michael P. O’Boyle, Zoe Hemond, Vernon L. Gay, Kelly Suter

**Affiliations:** ^1^Department of Biology, University of Texas San Antonio, San Antonio, TX, USA; ^2^Department of Cell Biology and Molecular Physiology, University of Pittsburgh School of Medicine, Pittsburgh, PA, USA; ^3^Neuroscience Institute, University of Texas San Antonio, San Antonio, TX, USA

**Keywords:** rat puberty, dendrite morphology, pubertal neuron activity, dendrite develop, GnRH neuron

## Abstract

Until the recent past, the search for the underlying drive for the pubertal increase in gonadotropin-releasing hormone (GnRH) hormone from the GnRH-containing neurons in the hypothalamus was largely focused on extrinsic factors. The most recent evidence however indicates changes in the structure of GnRH neurons themselves may contribute to this fundamental event in development. Based on our studies in males, dendritic architecture is not static from birth until adulthood. Instead, dendrites undergo a dramatic remodeling during the postnatal period which is independent of testosterone and occurs before the pubertal increase in GnRH release. First, the number of dendrites emanating from somata is reduced between infancy and adulthood. Moreover, a dendrite of adult GnRH neurons invariability arises at angle of 180°from the axon as opposed to the extraordinary variability in location during infancy. In fact, in some neurons from infants, no dendrite even resides in the adult location. Thus, there is a spatially selective remodeling of primary dendrites. Secondly, dendrites of GnRH neurons from infants were highly branched prior to assuming the compact morphology of adults. Finally, other morphological aspects of GnRH neurons such as total dendritic length, the numbers of dendrite branches and the lengths of higher order branches were significantly greater in infants than adults, indicating a consolidation of dendritic arbors. Activity in multi-compartment models of GnRH neurons, suggest the impact of structure on neuronal activity is exerted with both active and passive dendrites. Thus, passive properties make a defining contribution to function. Accordingly, changes in morphology alone are likely to have functional consequences for the pattern of activity in GnRH neurons. Our findings suggest structural remodeling of dendrites during the postnatal period likely facilitates repetitive action potentials and thus, GnRH release at the time of puberty.

Puberty and its demarcation of the beginning of the reproductive lifespan is one of the most dramatic transitions in physiology. In virtually all vertebrate species, puberty depends on an adult pattern of pulsatile secretion of gonadotropin-releasing hormone (GnRH) from the GnRH-containing neurons of the hypothalamus. Accordingly, puberty ultimately reflects a shift in the output of the so-called GnRH “pulse” generator. Thus, while puberty results in a host of physiological and behavioral features (Romero et al., 2002), we consider the onset of puberty, in the context of this review, as a discreet, centrally mediated event culminating in the ability to reproduce with a focus on the control of puberty in the male.

## Mechanisms Which Account for the Onset of Puberty: The Usual Suspects

It is generally accepted that each pulse of luteinizing hormone (LH) from the anterior pituitary is preceded by a pulse of GnRH from the hypothalamus. Therefore, changes in LH secretion have been used to track the developmental time course of the initiation of reproduction. The earliest studies differed as to the age of the onset of puberty; differences which most likely reflected the strain of rats that had been studied. Nonetheless, an over-arching consensus emerged that gonads were a critical component of the pubertal control mechanism in rodents. Castration resulted in a sustained elevation of LH with a magnitude of about two thirds of LH levels in castrated adult males (Moore and Price, 1932; Goldman et al., 1971; Eldridge et al., 1974; Negri and Gay, 1976). Moreover, the response to gonadectomy was relatively rapid: circulating levels of LH were elevated within 24 h of castration. The response is similar to that of adult male rats following castration (Eldridge et al., 1974). This post-castration response could be evoked at any point during the prepubertal period by removal of the gonads (Goldman et al., 1971; Eldridge et al., 1974). However, immature males exhibited a heightened sensitivity to exogenous steroids relative to adult males (Ramirez and McCann, 1965; Bloch et al., 1974; Eldridge et al., 1974; Döhler and Wuttke, 1975; Negri and Gay, 1976). Taken together, these findings suggested the GnRH pulse generator had the potential to function in male rodents at the time of birth but was held in check by a heighted sensitivity to the low levels of testosterone (T) produced and released by the neonatal testes. Accordingly, the onset of puberty was proposed to be controlled by a “gonadostat” whose ability to inhibit hormone secretion from GnRH neurons decreased during development (Moore and Price, 1932).

While the concept of the gonadostat has formed the linchpin in understanding control of puberty in rodents, the composition of the gonadostat remains unknown. Nonetheless, from an operational standpoint the gonadostat must communicate with the GnRH neurons involved in pulsatile hormone secretion and exhibit a sensitivity to gonadal steroids which is altered during maturation of the hypothalamus. In the context of inhibition of GnRH neurons, an inhibitory neurotransmitter system would be activated by gonadal steroids and inhibit either the activity of individual GnRH neurons or prevent coordination of GnRH neurons such that GnRH release is limited so as to result in only a slow frequency of GnRH release. Alternatively, the gonadostat may exert its effect by a lack of excitation. In this sequel, gonadal steroids might restrain the activity of a stimulatory influence on GnRH neurons or prevent communication between GnRH neurons and a stimulatory influence.

A wide variety of neurotransmitters have been implicated in either inhibition or lack of excitation of GnRH neurons during prepuberty. Catecholamines and opioidergic systems appear to inhibit GnRH neurons prior to puberty. Conversely, GABA, excitatory amino acids and serotonin have all exhibited stimulatory effects in prepubertal males (Moguilevsky and Wuttke, 2001). A prime candidate for activation of GnRH neurons as a prolog to puberty is the kisspeptin-containing neurons. During the past decade it has been demonstrated that a kisspeptin receptor (GPR54) is co-localized with GnRH neurons. The number of kisspeptin mRNA-expressing neurons increased gradually across the prepubertal interval (Bentsen et al., 2010). In male rats, the arcuate nucleus appears to contain kisspeptinergic neurons which may be involved in the onset of puberty (Bentsen et al., 2010; Takumi et al., 2011; Desroziers et al., 2012). Likewise, quantitative or qualitative differences in the synaptic input to GnRH neurons might well contribute to the increase in GnRH neuronal activity at the time of puberty (see Ebling, 2005 for review). This process might involve removal of inhibitory synaptic input, increasing stimulatory input, or both, especially since GnRH secretion can be influenced by almost all major monoamine and amino acid neurotransmitters (Todman et al., 2005). Direct measurements of post-synaptic modifications on GnRH neurons indicate that the synaptic input to GnRH neurons increases between prepuberty and adulthood in male rats. It is of interest that some prepubertal neuronal profiles were contacted by GnRH immunoreactive nerve terminals, while no such interactions were observed in the GnRH neurons of the adults (Witkin and Romero, 1995). The significance of these GnRH–GnRH neuronal interactions remains obscure. Nonetheless, both changes in afferent activity as well as retraction or imposition of synapses on GnRH neurons may all contribute to gonadostatic control.

## The Onset of Puberty is Relatively Rapid

Independent of the precise composition of the gonadostat, circulating levels of LH initially suggested that the onset of puberty reflected a gradual release of its control over GnRH secretion. However, recent direct sampling of the decapeptide demonstrated that the increase in GnRH secretion at the time of puberty occurs over a relatively short interval. In male rats, a slow frequency of GnRH pulses exists until about 45–46 days of age. Subsequently, the frequency of GnRH pulses abruptly increases at 47–48 days of age (Harris and Levine, 2003). Thus, the adult-like pattern of GnRH release emerges within 24–48 h of the time at which only prepubertal GnRH secretion was detected. Parenthetically, a similar rapid shift occurs in agonadal rhesus monkeys (Suter et al., 1998). Therefore, the pubertal increase in activity of GnRH neurons does appear to be a discreet event as opposed to a protracted event in males, at least when examined at the level of hormone secretion.

## Developmental Changes in Morphology and Topology of GnRH Dendrites: Not Your Usual Suspects

While changes in the activity of afferent inputs have been well-considered, our own studies suggest that alterations in morphology of the GnRH neuron may also contribute to the onset of puberty. Some of the earliest studies of GnRH morphology described a prepubertal transformation from “smooth” GnRH neurons to an “irregular” cell type due to spine-like processes extending from the cell membrane. It is known that dendritic spines are the location of excitatory synaptic input (Gray, 1959). Thus, this change in morphology would increase the area of specialized synaptic contact in the period just prior to puberty (Wray and Hoffman, 1986).

**Figure 1:**
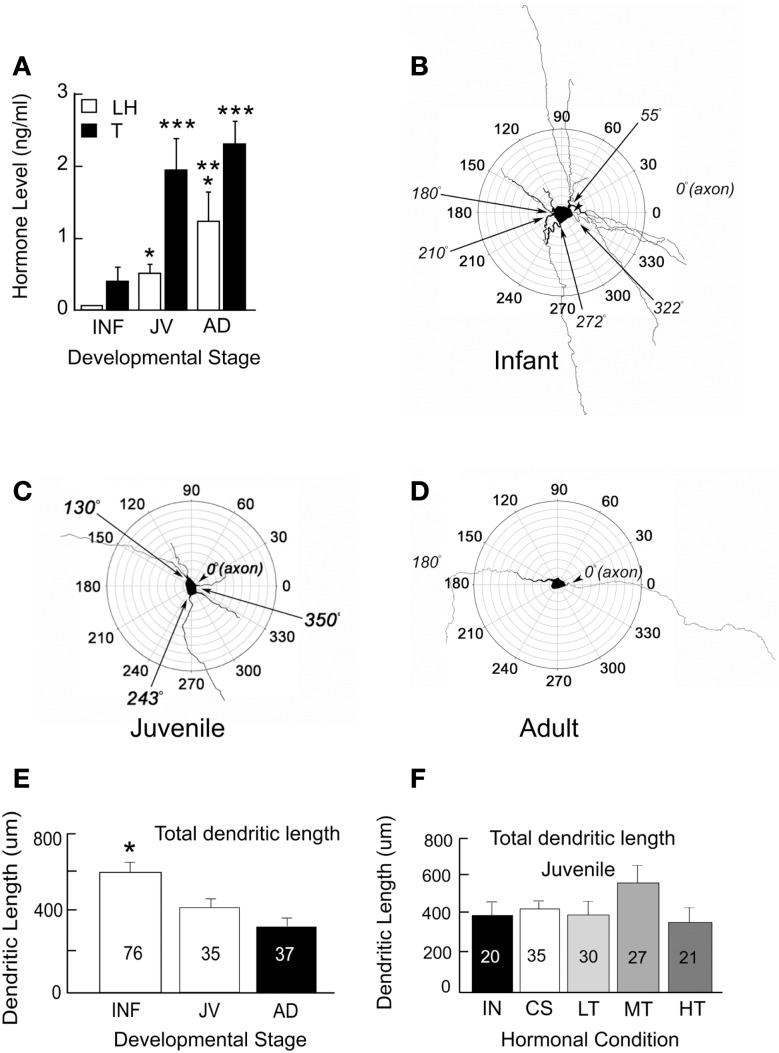
**Circulating levels of hormones (A) and polar plots of GnRH neurons from infant (INF, B), juvenile (JV, C), and adult (AD, D) male rats**. Single asterisks indicate LH levels that are significantly higher than those in INF. Double asterisks indicate LH levels that are higher than those in INF and JV. Triple asterisks indicate T levels that are higher than those in INF. Multiple primary dendrites emerge from GnRH somata during infancy. A majority of these dendrites are no longer present in juveniles and adults. Total dendritic length decreases throughout sexual maturation **(E)** but does not appear to depend on levels of T **(F)**. Males at about 43 days of age have the same total dendritic lengths whether they have testes (INT), were castrated (CS), or castrated and treated with low (LT), medium (MT), or high levels (HT) of T. Numbers of neurons are indicated on each bar. Figure is used from Ybarra et al. (2011). Copyright 2011, The Endocrine Society.

For an analysis of the structure and age-related changes, we studied the morphology of GnRH neurons in male transgenic rats from infancy to adulthood. These neurons had been filled with biocytin during electrophysiological recordings (Ybarra et al., 2011). Based on circulating levels of LH and T, the onset of puberty occurs prior to about 43 days of age (Figure 1A). This is an earlier age than when Harris and Levine (2003) detected an increase in pulsatile GnRH. Their study however used the Sprague Dawley rat strain as opposed to the Wistar strain which is the background strain for the GnRH-GFP transgenic rats used in our studies (Kato et al., 2003).

The maturation of GnRH neurons exhibits a progression from a complex structure during infancy (Figure 1B) to one which is both more compact and less complicated in juveniles and adults (see Figures 1C,D). Between infants and adults, somatic area decreased by almost half. Maturation of dendrites was characterized by a progression from a stellate conformation with many primary dendrites to one with generally a single dendrite. This consolidation of GnRH dendrites during the prepubertal period resulted in reduction in total dendritic length (sums of all branch lengths) by about half (Figure 1E) reflecting the loss of multiple primary dendrites of infancy. In infancy, some primary dendrites emerged from somata near the same location as the axon. Others were present across the entire circumference of somata. The simplification of the dendritic arbor leads to the configuration of the adult neuron in which one dendrite and one axon emerge from each GnRH somata with a consistent axo-dendritic angle of 180° between the two.

The above findings provide support for the hypothesis that critical features of the dendrites of GnRH neurons continue to differentiate postnatally. The proximate cause of developmental alterations in GnRH dendrites remains speculative. To ask whether alterations in dendritic structure are due to T, we castrated male rats at 25 days of age. Males were then either treated with one of three levels of T or left without T replacement. Morphological features of their dendrites were then examined at the time puberty would have been anticipated in our rat colony. Figure 1F summarizes dendritic parameters of interest in T-treated males and compares them to intact and castrated male rats of the same age. Altering the level of T throughout the peripubertal period had no impact on any of these dendritic features at the time of puberty.

The finding that both local and global alterations in GnRH dendrites are independent of gonadal steroids, suggests the possibility that late, post-natal changes in GnRH dendrites may in part reflect an initial developmental program but subsequent remodeling depends on hypothalamic influences. The notion that morphological changes in dendrites of GnRH neurons are directed in part by a developmental program is supported by observations in the GT1-7 cell line, a model for GnRH neurons (Mellon et al., 1990). In culture, GT1-7 cells have atypical shapes and extend thin processes (Liposits et al., 1991; Maggi et al., 2000) much like immature GnRH neurons. Interestingly, after transplantation into the hypothalamus, the dendritic arbor becomes more typical of the adult GnRH neuron (see Figure 5; Silverman et al., 1992). Taken together this suggests that the initial dendritic arbor of GnRH neurons reflects an intrinsic program but subsequent elaboration of dendrites and or retraction of early dendrites occurs based on signaling that arises in the hypothalamic setting.

**Figure 2:**
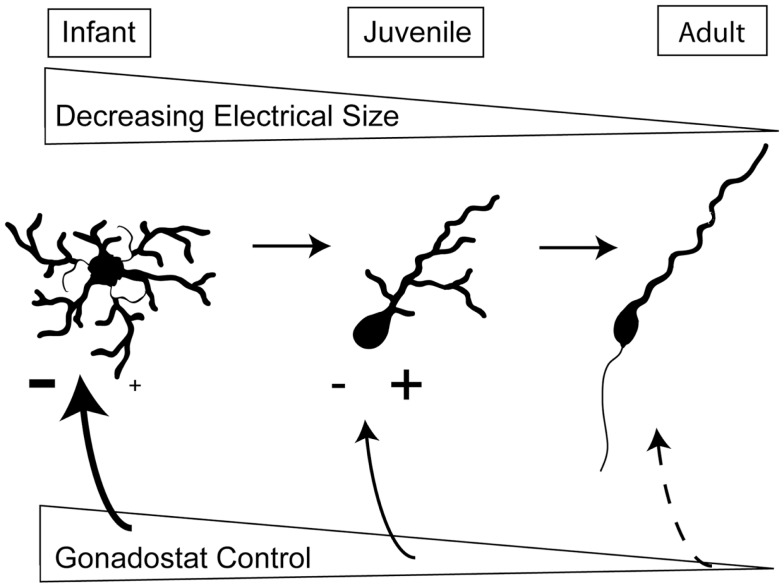
**Changes in intrinsic and extrinsic control of GnRH neurons across sexual development**. During prepuberty, the larger area of GnRH neurons limits their responsiveness to synaptic input. Additionally, GnRH neurons have fewer spines, the location of excitatory synapses. The stimulation from kisspeptin-containing neurons also appears to be limited. By the juvenile phase of development, the dendritic arbor has decreased, the number of spines has increased, and kisspeptin inputs are likely stimulating GnRH neurons. In adulthood, the final morphology has been achieved and the adult-like synaptic drive is present.

Figure 2 summarizes the intrinsic and extrinsic changes in GnRH neurons which likely contribute to the onset of puberty. First, the decrease in somatic size as well as the simplification of the structure of dendrites reduces plasma membrane area and therefore, decreases the effective electrical size of GnRH neurons. This makes neurons more responsive to changes in synaptic input. Since T does not appear to influence these structural changes, the consolidation of dendritic arbors is probably not part of the gonadostat. Instead, we suggest that the morphological changes represent a gonad-independent control mechanism in rodents. Secondly, the level of excitation to GnRH neurons increases while inhibitory influences decrease. Either of these changes could be influenced by gonadal steroids and therefore, comprise the gonadostat. Independent of the specific alterations in extrinsic control, morphological changes in the GnRH neurons themselves will determine the effectiveness of the gonadostat.

## Conflict of Interest Statement

The authors declare that the research was conducted in the absence of any commercial or financial relationships that could be construed as a potential conflict of interest.
